# Video Lecture Capture in Pharmacy Education: Insights From the Pandemic Experience

**DOI:** 10.7759/cureus.73649

**Published:** 2024-11-13

**Authors:** Farhat N Hussain, Reem Al-Mannai, Abdelali Agouni

**Affiliations:** 1 Department of Pharmaceutical Sciences, College of Pharmacy, QU Health, Qatar University, Doha, QAT; 2 Department of Clinical Pharmacy and Practice, College of Pharmacy, Qatar University, Doha, QAT

**Keywords:** covid-19, distance learning, pandemic, pharmacy education, technology-enhanced learning, video lecture capture

## Abstract

The COVID-19 pandemic prompted an abrupt and widespread transition to remote learning, compelling higher education institutions to swiftly adjust to novel modalities. This study examines the post-pandemic insights and benefits of technology-enhanced learning (TEL), particularly archived video lecture capture systems, in facilitating undergraduate pharmacy education during and beyond the pandemic. Through a quantitative analysis of archival lecture views, we sought to identify critical elements that enabled a successful transition to remote learning, providing insights into sustainable educational methods for future disruptions. Our analysis indicated a substantial rise in weekly views of lecture archived recordings during the pandemic, with Spring 2020 achieving 452 views compared to 291 in Spring 2019. Usage surges transpired during weeks 11-13 of Spring 2020, aligning with the sudden transition to remote learning and a temporary postponement of assessments. A comparative analysis of archived lectures from Spring 2019 (delivered before the pandemic) at two intervals - November 2019 and September 2020 - revealed a significant rise in views following the pandemic. The temporal analysis of lecture archives access indicated that students sustained their after-hours watching patterns, with a peak occurring between 6 PM and 9 PM, particularly before major exams. Nevertheless, the frequency of access significantly increased after the pandemic.

This study emphasizes the strategic importance of video lecture recording in enhancing resilience within higher education institutions, facilitating students' learning continuity, and equipping institutions for possible future disruptions. These technologies facilitate flexible, autonomous learning, catering to diverse student needs and learning styles. They also support faculty professional development by encouraging reflective practice and data analysis to improve teaching methods. Furthermore, video recording facilitates instructor collaboration and acts as a vital resource for ongoing education, allowing students to revisit complex topics. Integrating TEL and video capture systems into the conventional academic framework enables higher education to improve its readiness and adaptability to uphold educational quality during unexpected occurrences.

## Introduction

The swift advancement of higher education has been significantly influenced by the incorporation of technology-enhanced learning (TEL) strategies [1], with video lecture capture (VLC) being a pivotal instrument [2,3]. Worldwide, TEL and VLC systems have gained popularity [4], with over 1,000 institutions documenting in excess of 100 hours of lectures monthly [5]. This growth highlights the acknowledgment of TEL as an essential element of contemporary educational methodologies, particularly as institutions address both existing and forthcoming issues that undermine conventional, face-to-face education methods. The COVID-19 pandemic exposed the inadequacies of exclusive reliance on in-person instruction and necessitated a swift transition to online and mixed learning modalities, underscoring the imperative for higher education systems to cultivate resilience and readiness for such disruptions in the future [6]. The rising frequency of health emergencies, such as the appearance of the monkeypox virus and novel virulent strains of COVID-19, underscores the necessity for flexible, resilient educational infrastructures that can sustain continuity amid unexpected disruptions [7].

The incorporation of TEL, especially via lecture video recording and archiving, has demonstrated advantages that extend beyond simple disaster readiness. VLC provides students with the opportunity to view recorded lectures at their convenience, facilitating a self-paced and flexible learning experience. Students can pause, rewind, and revisit intricate information, enabling them to enhance their comprehension of difficult ideas and promote autonomous learning. Moreover, VLC resolves a critical challenge in education, allowing students who have been absent due to illness, familial responsibilities, or other reasons to recuperate missing material without lagging behind. This flexibility aids students in achieving learning outcomes and fosters a more inclusive educational environment that addresses varied student needs and learning preferences [8-12].

In addition to their direct effects on students, lecture video recordings and TEL provide unique benefits for professors and enhance overall teaching quality. Lecture video recordings function as an invaluable resource for peer observation and feedback, enabling faculty members to examine each other's teaching methodologies and obtain constructive critiques to enhance their methods. Additionally, educators can conduct self-evaluations by reviewing their own recordings, thereby acquiring insights into their presentation abilities, timing, and clarity. This form of reflective practice is essential for ongoing professional development, promoting a culture of enhancement inside organizations. Archived lectures are essential for faculty training; they offer new faculty members exemplary lectures, facilitate the alignment of teaching methodologies across courses, and guarantee uniformity in curriculum delivery, particularly in disciplines necessitating standardized instruction. Furthermore, these archives serve as a resource for unforeseen absences, guaranteeing continuity when a faculty member is unable to conduct a scheduled session, thus minimizing dependence on replacement instructors and preserving instructional integrity [13,14].

The educational benefits of lecture-captured content encompass wider pedagogical innovations, such as the flipped classroom model, wherein students review lecture material before class, thereby reserving in-class time for enhanced interactive and collaborative learning. This methodology fosters active learning since students arrive equipped to engage in discussions, resolve problems, and implement information in practical contexts. This method corresponds with contemporary pedagogical best practices that prioritize student-centered, active learning settings and has demonstrated the ability to increase student engagement and enrich learning. The capacity to document and store lectures facilitates this model, enabling students to access essential content beyond the classroom and concentrate in-person sessions on reinforcing and applying their acquired knowledge.

Nonetheless, utilizing lecture video recordings has certain complications. Concerns over diminished attendance are prevalent since several faculty members fear that easily accessible recordings may deter students from participating in live lectures. Some studies indicate a decrease in attendance associated with VLC availability, while others reveal no significant effect, implying that attendance may be affected by criteria beyond mere lecture availability, including class participation and perceived relevance [15-17]. Concerns exist over excessive dependence on recorded content, which may diminish active participation in live sessions; nevertheless, these risks can frequently be alleviated through deliberate course design and engagement tactics. Furthermore, equitable access to TEL resources continues to pose a barrier, since not all students may possess equivalent access to essential technology and internet connectivity. It is essential to guarantee that TEL efforts are inclusive and accessible to all students in order to effectively harness the advantages of modern educational tools.

The current study investigates the influence of the COVID-19 pandemic on archived lecture video recordings utilization in higher education, concentrating on the undergraduate pharmacy degree at Qatar University. In March 2020, the Government of Qatar mandated the cessation of in-person classes to mitigate the virus's transmission, leading to a sudden transition to online instruction [18]. The College of Pharmacy at Qatar University, with over 10 years of experience in TEL and VLC use, was adequately equipped to shift to a completely remote approach. The college was one of the initial institutions at the university to systematically implement video recordings of live lectures, utilizing the Echo360 system (Youngstown, OH: Echo360 Inc.) to record audio and video lectures, integrate PowerPoint presentations (Redmond, WA: Microsoft Corp.), and facilitate convenient access via the Blackboard virtual learning environment (Washington, DC: Blackboard Inc.). This platform enables students to access lectures from both current and prior years until graduation, while faculty can monitor and evaluate watching patterns to gauge student engagement with the recorded material [13,14,18].

This study expands on our previous study regarding the use of archived lecture video recordings in professional pharmacy courses across successive academic years, specifically during the spring 2019 semester before the pandemic [13,14,18]. This study intends to examine alterations in viewing habits before and during the pandemic, emphasizing the heightened dependence on VLC archives as an alternative to in-person lectures. We examine factors such as the time of day, week of the semester, and assessment frequency to comprehend the changes in viewing behaviors resulting from the shift to online learning. This method allows us to assess the contribution of VLC to maintaining student learning continuity and to pinpoint future enhancements in TEL integration within the pharmacy curriculum.

This research enhances the broader discourse on the role of TEL in strengthening the resilience of higher education systems. This study analyses the usage of archived lecture video recordings and adaptation during a period of substantial disruption, offering insights into the effectiveness of TEL infrastructure in sustaining educational continuity and quality. Our findings can assist higher education institutions in enhancing their TEL policies, assuring preparedness for future disruptions. The use of TEL enhances institutional readiness for emergencies while promoting inclusive, adaptable, and efficient learning environments that benefit both students and faculty. The findings of this study highlight the necessity of integrating TEL into educational frameworks, promoting its continuous application as a means to enhance educational outcomes, facilitate faculty development, and foster resilience against future shocks to conventional learning paradigms.

## Materials and methods

Study population and methods of data collection

The number of VLC viewings per lecture was documented for undergraduate pharmacy courses at the College of Pharmacy, Qatar University, during the spring semester of the 2018/2019 academic year (designated as spring 2019) at two following intervals: November 2019 and September 2020. Our study encompassed 15 courses for analysis: four courses from professional year 1 (P1), five courses from professional year 2 (P2), four courses from professional year 3 (P3), and two courses from professional year 4 (P4). Table 1 delineates the course titles and corresponding numbers. We examined a total of 213 lecture archives, and viewing statistics were presented as average views per lecture. We then compared the data from September 2020 for these courses to previously extracted data from November 2019 to assess the impact of the emergency transition to distance learning during the spring semester of the 2019/2020 academic year (19AY), hereafter referred to as spring 2020 [13]. Courses lacking data for November 2019 or those that were laboratory-based were omitted from the comparison conducted in September 2020. In certain analyses, lectures were categorized into pre- and post-suspension of in-person classes subsequent to the emergency transition to remote learning (before and after 9 March 2020 or week 11).

**Table 1:** Spring semester courses that are included in the analysis of VLC views. VLC: video lecture capture

Course name	Course code	No. of lectures analyzed	No. of students enrolled
Year 1 (P1)			
Medicinal chemistry II	PHAR201	23	34
Pharmaceutics I	PHAR210	25	33
Foundations of pharmacology and pharmacotherapeutics II	PHAR221	10	33
Pharmacy and healthcare II	PHAR231	8	33
Year 2 (P2)			
Pharmacy research, evaluation and presentation skills II	PHAR306	9	37
Pharmaceutics Ill	PHAR311	13	35
Pharmacokinetics II	PHAR317	12	35
Interpretation of lab data II	PHAR360	9	33
Integrated courses (IC) (pathophysiology, pharmacology, and pharmacotherapeutics)	IC2	40	33
Year 3 (P3)			
Pharmacy research, evaluation, and presentation skills IV	PHAR406	7	30
Pharmacognosy alternative/complementary treatments	PHAR425	7	30
Pediatrics/geriatrics	PHAR485	6	32
Integrated courses (IC) (pathophysiology, pharmacology, and pharmacotherapeutics)	IC4	28	31
Year 4 (P4)			
Pharmacoeconomics	PHAR525	9	16
Pharmacy management	PHAR535	7	16

For certain analyses, the aggregate number of views of Echo360 archives per week and per hour of the day during spring 2019 and spring 2020 were documented and compared to each other. Furthermore, for spring 2020, total views per week or day were compared between the periods preceding and succeeding the suspension of in-person classes due to the emergency transition to distance learning (before and after 9 March 2020, or week 11).

Three investigators (FN, AA, RM) were provided access to view statistics of archived lecture video recordings using the Echo360 video management application. Two investigators (FN, RM) examined each spring course in the undergraduate pharmacy program and recorded viewing statistics in Google Sheets (Mountain View, CA: Google LLC). Data were accessible for each course and lecture, with viewing statistics provided for most courses. The data obtained for views per lecture for spring 2019 courses in September 2020 were subsequently compared to the data acquired in November 2019. The data were checked and analyzed by AA before being aggregated for statistical analysis. The mean number of views per course and level was documented and compiled. We documented the weekly and hourly view counts and summarized them for both spring 2019 and spring 2020.

Ethics

No interaction with human subjects occurred for study purposes; thus, ethical review was unnecessary. Permission to utilize the statistics on the use of archived lecture video recordings for the publication of these results was granted by the college administration.

Statistics and data analysis

Data are presented as views per VLC, represented as mean±standard error of mean (SEM). Statistical analyses were conducted utilizing either a paired t-test or a one-way analysis of variance (ANOVA), accompanied by Tukey’s post hoc test when applicable. The normality of the data was assessed using the D’Agostino, Pearson, and Shapiro-Wilk tests on each occasion. P≤0.05 was deemed statistically significant. Analysis was performed utilizing GraphPad Prism 7.0e software (San Diego, CA: GraphPad Software, LLC).

## Results

Impact of COVID-19 pandemic on archived VLC views

**Figure 1:**
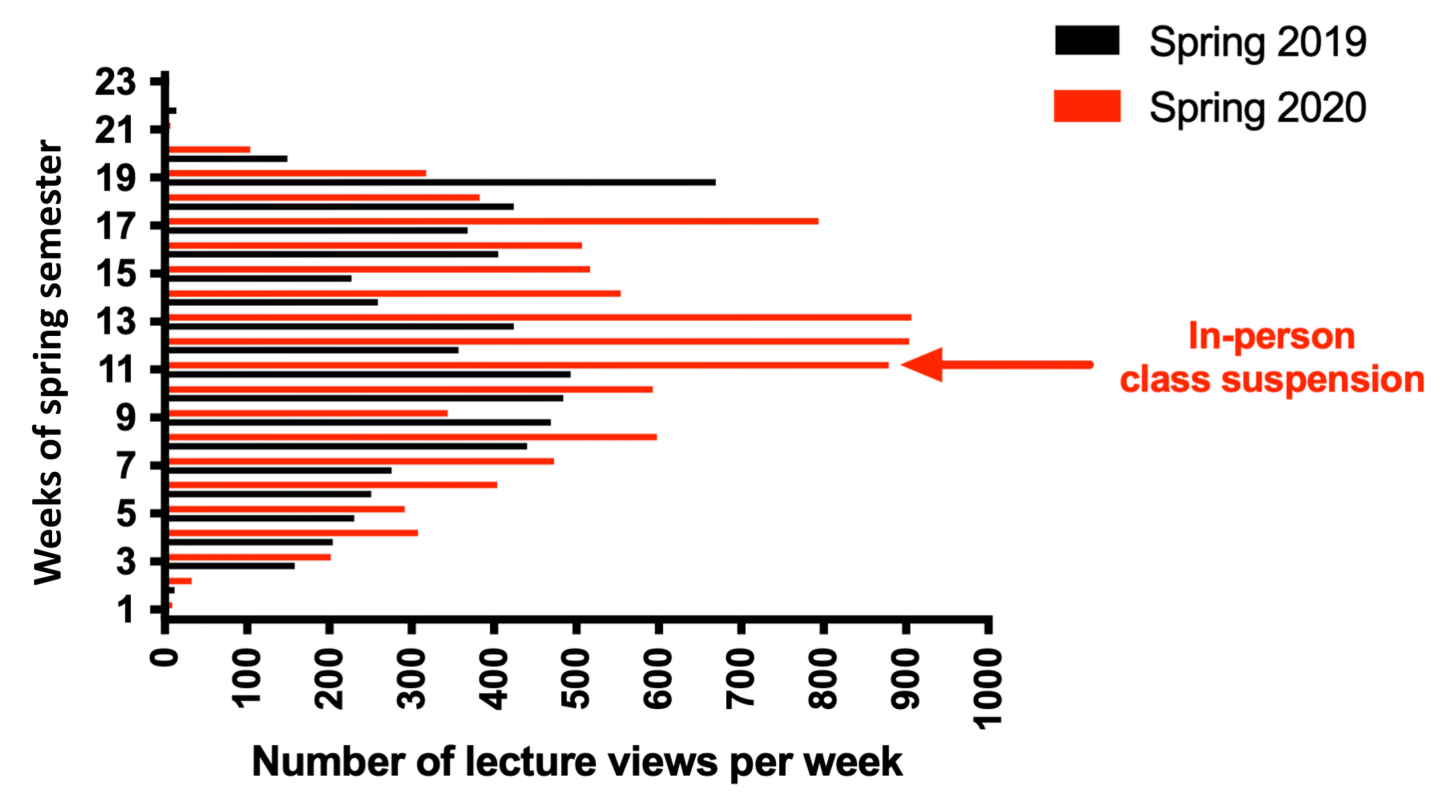
Weekly comparison of lecture archive views in spring 2019 versus spring 2020. The number of lecture archive views per week is shown for spring 2019 (black bars) and spring 2020 (red bars).

Figure 1 depicts the number of lecture capture views per week for spring 2019 (black-colored bars) and spring 2020 (red-colored bars). The total number of Echo360 recording views in spring 2020 (9,135 views) was nearly 45% higher compared to spring 2019 (6,316 views). The median number of views in spring 2020 (383 views) was higher compared to spring 2019 (259 views). The average number of views per week in spring 2020 (397 views) was also higher compared to spring 2019 (274 views). The maximum number of views per week observed in spring 2020 was 907, while in spring 2019 this culminated at 669 views.

We analyzed the average weekly views documented in spring 2020 following the cessation of on-campus classes (post-week 11) and compared them to the average weekly views recorded during the corresponding period in spring 2019. During this period, the average weekly views were more in spring 2020 (452 views) than in spring 2019 (291 views) (Figure 1).

Figure 1 illustrates a significant increase in views during weeks 11 (879 views), 12 (904 views), and 13 (907 views) of spring 2020 (from March 11 to March 29, 2020), in contrast to preceding weeks, coinciding with the onset of in-person class suspension and the sudden transition to remote learning on March 9, 2020. The total view count fell by around 40% during weeks 14-16 due to the commencement of live sessions and online instruction at the college; however, the average weekly views remained elevated compared to the period preceding the suspension of in-person classes (before week 10). Nonetheless, a significant surge in views occurred during week 17 (20-26 April 2020) with 794 views, coinciding with a substantial volume of assignments and midterms, and immediately preceding the commencement of final exams in week 18 (Table 2). Conversely, the view count in spring 2019 exhibited the most significant weekly surge in week 19, reaching 669 views, coinciding with the first week of final examinations in spring 2019 (Figure 1).

**Table 2:** Mapping of the number of assessments per week to the number of lecture archive views.

Weeks (spring 2020)	No. of assessments	Remarks		No. of lecture archive viewings
Week 1	0	-	9	
Week 2	0	-	33	
Week 3	0	-	202	
Week 4	1	-	308	
Week 5	1	-	292	
Week 6	3	-	404	
Week 7	6	-	473	
Week 8	9	-	598	
Week 9	9	-	344	
Week 10	0	Mid-spring semester break	593	
Week 11	0	Suspension of on-campus classes	879	
Week 12	0	-	904	
Week 13	0	-	907	
Week 14	0	-	554	
Week 15	12	Objective structured clinical exam (OSCE)	517	
Week 16	15	-	507	
Week 17	18	-	794	
Week 18	15	Start of final exams	383	
Week 19	18	-	318	
Week 20	3	-	104	
Week 21	0	-	7	
Week 22	0	-	4	
Week 23	0	-	1	

Comparison of the average number of views per session for spring 2019 archived lectures at two-time points between November 2019 and September 2020

After the suspension of in-person classes on campus, all teaching activities, including lectures, were delivered remotely using a variety of live teaching platforms, such as Blackboard Collaborate Ultra (Washington, DC: Blackboard Inc.), Microsoft Teams (Redmond, WA: Microsoft Corp.), WebEx (San Jose, CA: Cisco Systems, Inc.), and Zoom (San Jose, CA: Zoom Video Communications, Inc.). Since video recording using the Echo360 system requires lectures to be delivered inside the classrooms, very few recordings were posted on Blackboard after March 9, 2020. Therefore, we postulated that the increase in the number of VLC views after the suspension of in-person classes may have been driven by an increased number of views of the previous academic spring 2019 semester archived lectures (Figure 1).

We have, therefore, compared the number of views for spring 2019 lectures at two-time points before and after the suspension of in-person classes - November 2019 (data were collected from a previously published report [13]) and September 2020. The average number of views per lecture was compared across 15 courses delivered in spring 2019 for all professional pharmacy years (P1, P2, P3, and P4) at these two-time points.

**Figure 2:**
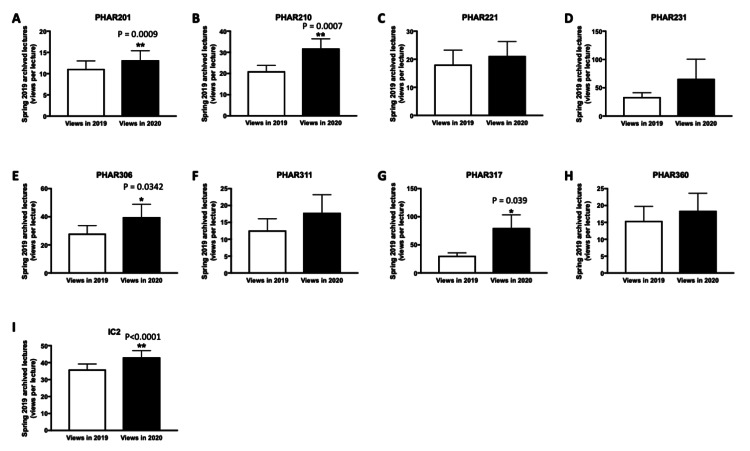
Comparison of the average views per lecture (P1 and P2 courses) for spring 2019 archived recordings at two-time points - November 2019 (2019 views) and September 2020 (2020 views). *P<0.05 was considered statistically significant compared to 2019 views **P<0.001 was considered statistically significant compared to 2019 views. (A-D) Views per lecture for spring 2019 lecture archives for P1 courses (PHAR201, PHAR210, PHAR221, PHAR231). (E-I) Views per lecture for spring 2019 lecture archives for P2 courses (PHAR306, PHAR311, PHAR317, PHAR360, IC2). The bar charts display the average number of views per lecture, presented as mean±SEM. Group comparisons were analyzed using a paired t-test.

Figures 2A-2I illustrate that the average viewership per lecture for spring 2019 archives has risen for both P1 courses (Figures 2A-2D) and P2 courses between November 2019 and September 2020 (Figures 2E-2I). The most substantial increases were noted for PHAR201, PHAR210 (P1 courses), PHAR306, PHAR317, and IC2 (P2 courses). Significantly, the viewership of the pharmacokinetics II (PHAR317) course surged by approximately 2.7 times, escalating from an average of 30 views per lecture in November 2019 to 80 views per lecture in September 2020 (p<0.05), reflecting an increase of over 50 views per lecture, equating to more than one view on average for each student enrolled in the course during spring 2020 (with 35 students enrolled in spring 2020) (Figure 2G).

**Figure 3:**
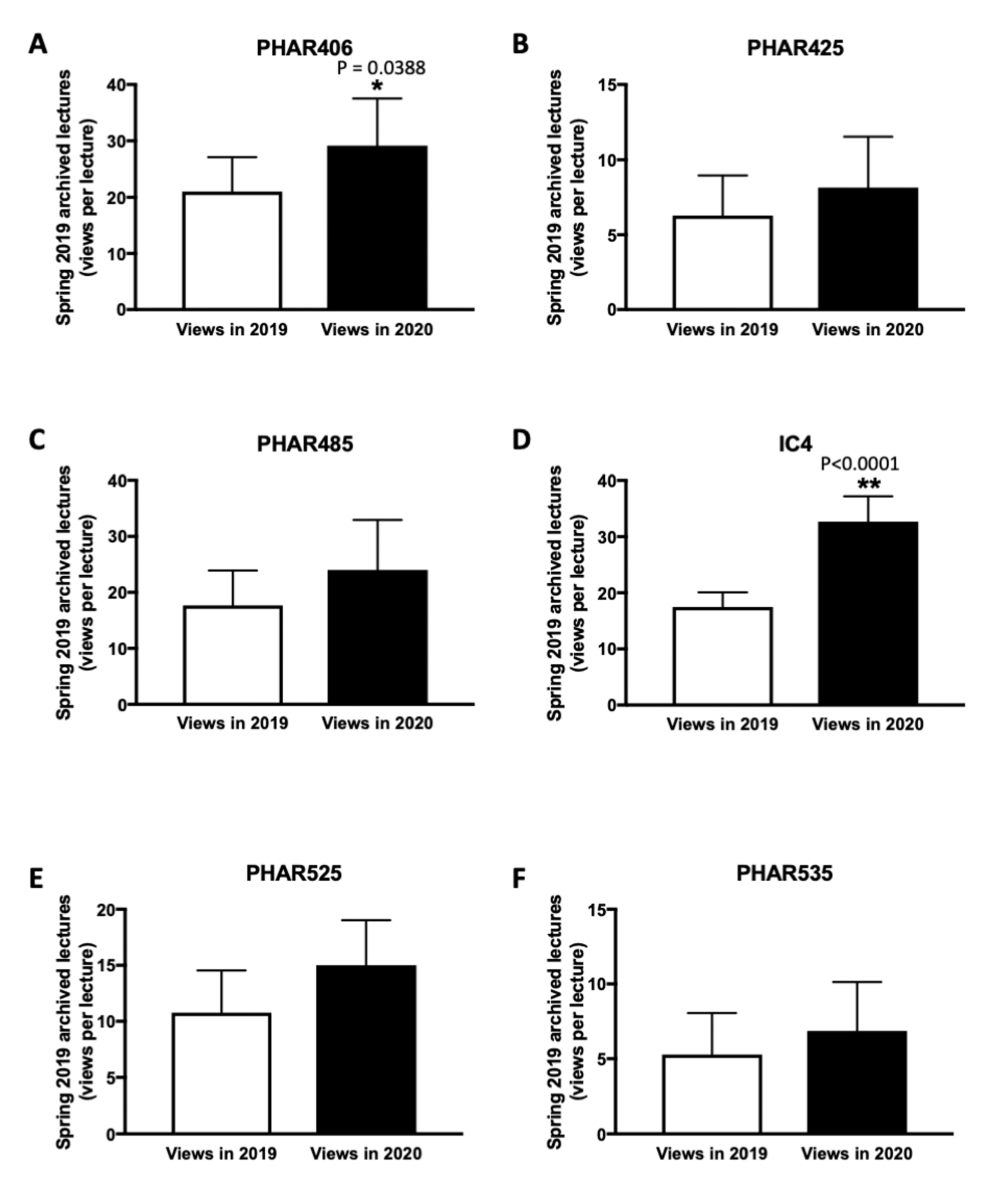
Comparison of the average views per lecture (P3 and P4 courses) for spring 2019 archived recordings at two-time points - November 2019 (2019 views) and September 2020 (2020 views). *P<0.05 was considered statistically significant compared to 2019 views. **P<0.001 was considered statistically significant compared to 2019 views. (A-D) Views per lecture for spring 2019 lecture archives for P3 courses (PHAR406, PHAR425, PHAR485, IC4). (E, F) Views per lecture for spring 2019 lecture archives for P4 courses (PHAR525, PHAR535). The bar charts display the average number of views per lecture, presented as mean±SEM. Group comparisons were analyzed using a paired t-test.

A similar pattern in the increase of the number of views was observed between November 2019 and September 2020 for P3 and P4 courses taken by more senior students (Figures 3A-3F). The most significant increases were observed for PHAR406, IC4 (P3 courses), and PHAR525 (P4 courses). Of note, for IC4 spring 2019 archives, the average number of views per lecture almost doubled between November 2019 and September 2020 (17-32 views per lecture; p<0.001) (Figure 3D).

The number of VLC views in spring 2020 was driven by an increased number of views for spring 2019 archived lectures after the suspension of in-person classes

To further harness the impact of in-person class suspension following the COVID-19 pandemic on viewings of Echo360 video lecture archives for courses delivered in the most recent academic year (spring 2019), we compared the average number of views per lecture for archived spring 2019 VLC for sessions delivered prior to and after the suspension of in-person classes (March 9, 2020).

**Figure 4:**
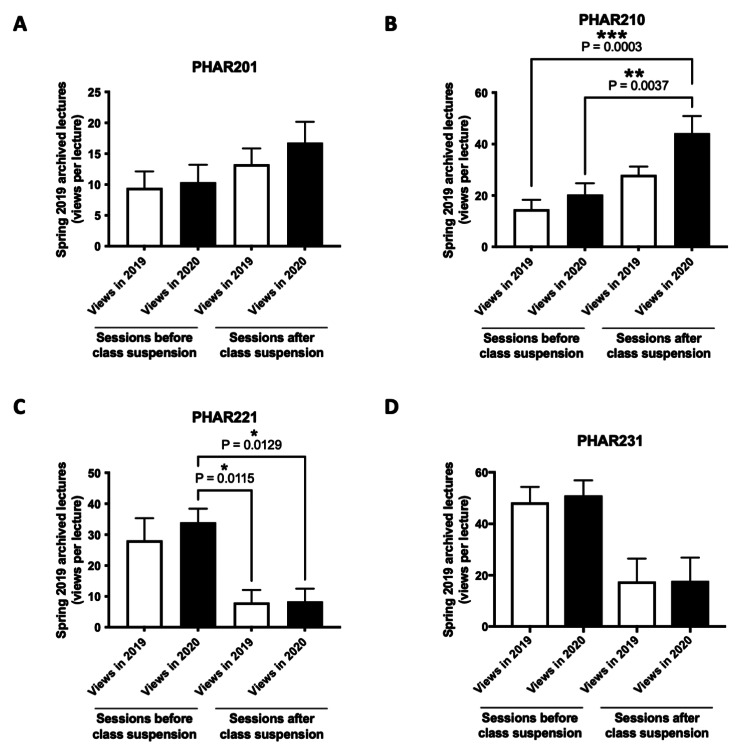
Comparison of the average views per lecture (P1 courses) for spring 2019 archived recordings before and after the suspension of in-person classes. *P<0.05 was considered statistically significant (indicated groups). **P<0.01 was considered statistically significant (indicated groups). ***P<0.001 was considered statistically significant (indicated groups). The average number of views per lecture for spring 2019 archived recordings, both before and after the suspension of in-person classes (lectures delivered before and after March 9, 2020, or week 11), was compared at two-time points - November 2019 (2019 views) and September 2020 (2020 views). (A-D), views per lecture for spring 2019 lecture archives for P1 courses (PHAR201, PHAR210, PHAR221, PHAR231). The bar charts display the average number of views per lecture, presented as mean±SEM. Group comparisons were performed using one-way ANOVA, followed by Tukey's post hoc test for multiple comparisons.

As shown in Figures 4A-4D, the average number of views per lecture for spring 2019 archives was significantly higher in September 2020 compared to November 2019 for P1 lectures delivered after the period of class suspension. The most significant differences were observed for PHAR201 (13-17 views per lecture) (Figure 4A) and PHAR210 (28-44 views per lecture (Figure 4B).

**Figure 5:**
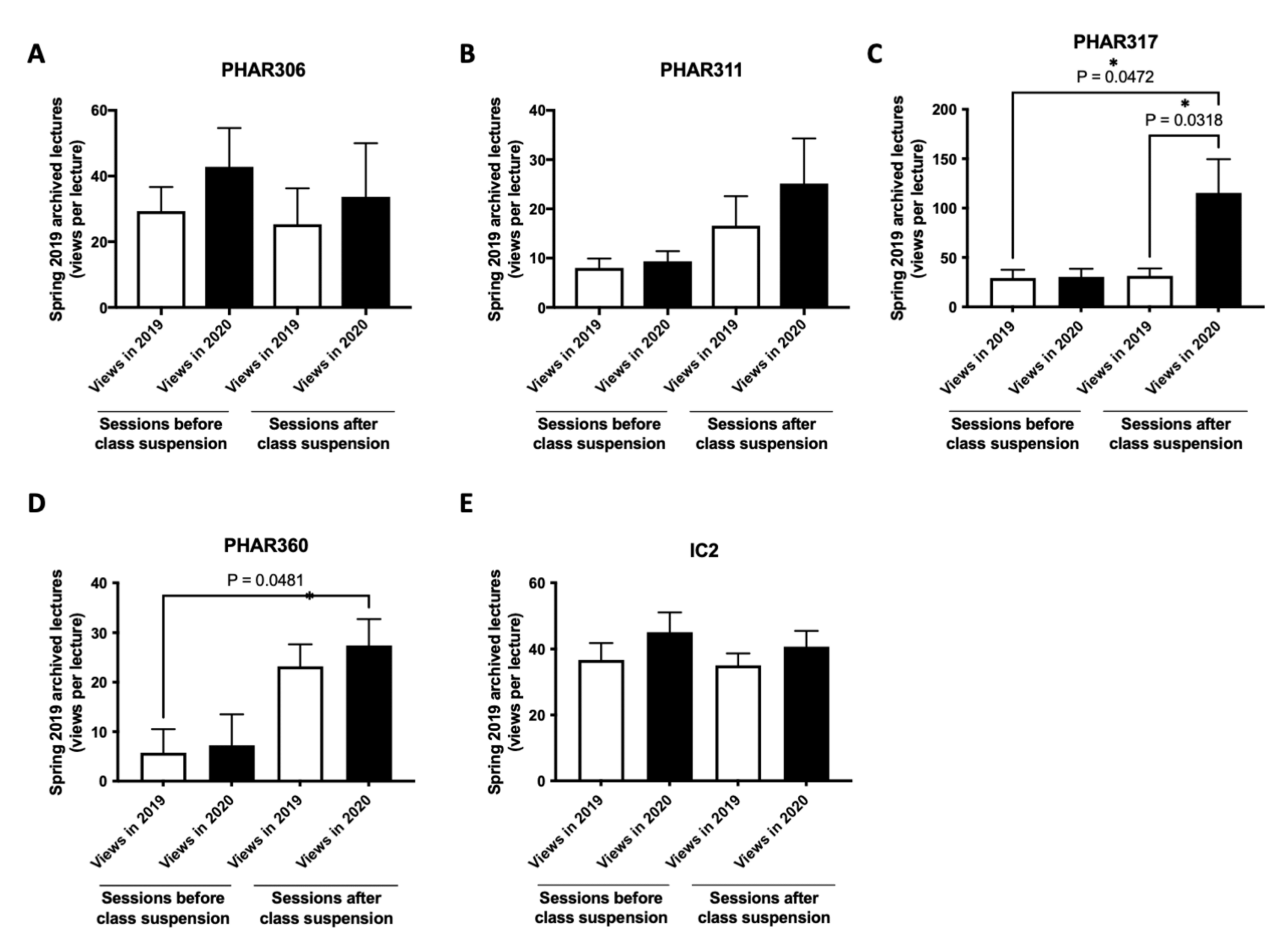
Comparison of the average views per lecture (P2 courses) for spring 2019 archived recordings before and after the suspension of in-person classes. *P<0.05 was considered statistically significant (indicated groups). The average number of views per lecture for spring 2019 archived recordings, both before and after the suspension of in-person classes (lectures delivered before and after March 9, 2020, or week 11), was compared at two-time points - November 2019 (2019 views) and September 2020 (2020 views). (A-E) Views per lecture for spring 2019 lecture archives for P2 courses (PHAR306, PHAR311, PHAR317, PHAR360, IC2). The bar charts display the average number of views per lecture, presented as mean±SEM. Group comparisons were performed using one-way ANOVA, followed by Tukey's post hoc test for multiple comparisons.

Similarly, P2 courses also saw an increase in the average number of views per lecture for spring 2019 archived lectures delivered after the suspension of in-person class suspension (Figures 5A-5E), with the highest changes observed for PHAR311 (16-25 views per lecture) (Figure 5B), PHAR317 (31-115 views per lecture) (Figure 5C) and IC2 (35-41 views per lecture) (Figure 5E).

**Figure 6:**
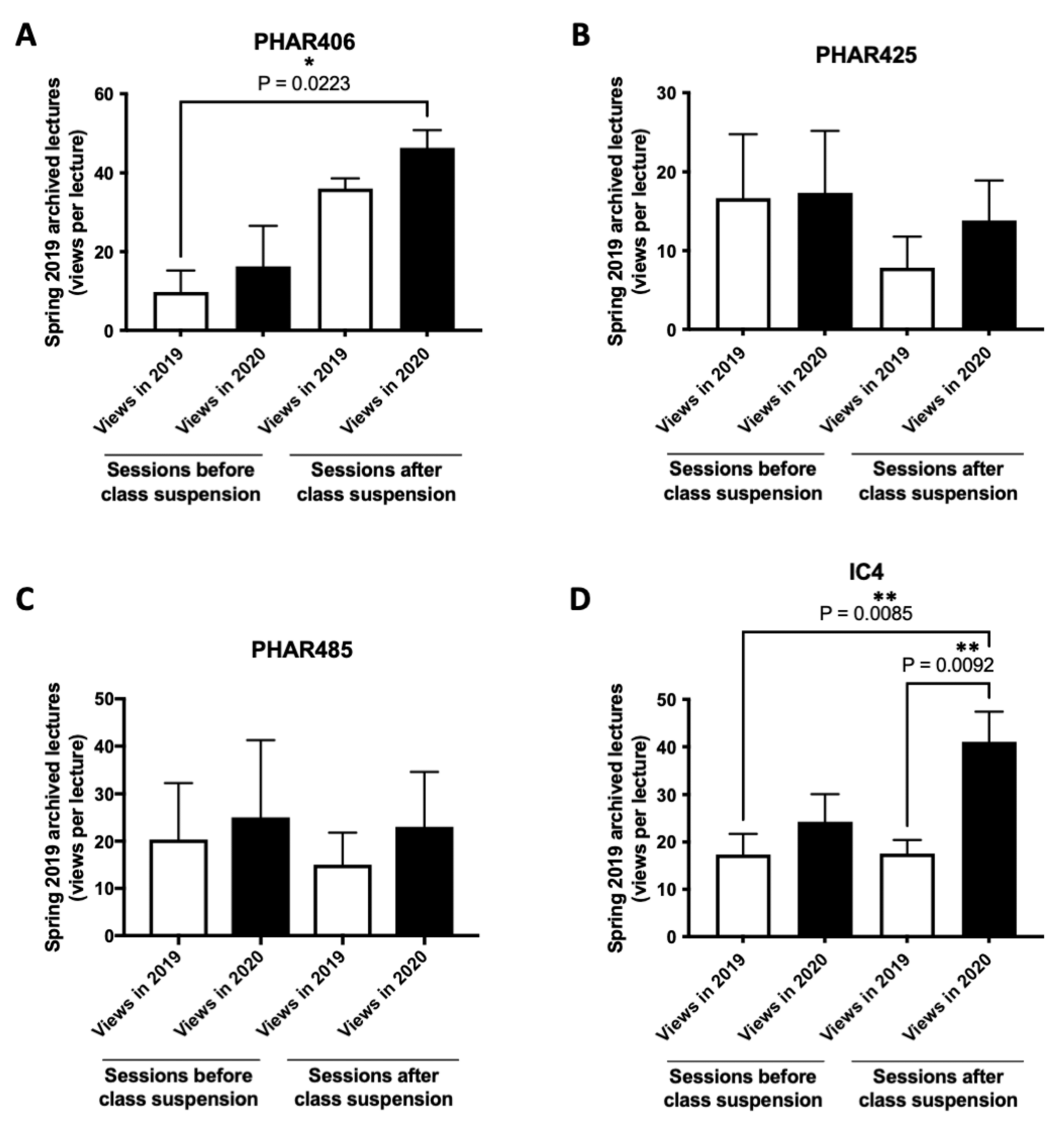
Comparison of the average views per lecture (P3 courses) for spring 2019 archived recordings before and after the suspension of in-person classes. *P<0.05 was considered statistically significant (indicated groups). **P<0.01 was considered statistically significant (indicated groups). The average number of views per lecture for spring 2019 archived recordings, both before and after the suspension of in-person classes (lectures delivered before and after March 9, 2020, or week 11), was compared at two-time points - November 2019 (2019 views) and September 2020 (2020 views). (A-D) Views per lecture for spring 2019 lecture archives for P3 courses (PHAR406, PHAR425, PHAR485, IC4). The bar charts display the average number of views per lecture, presented as mean±SEM. Group comparisons were performed using one-way ANOVA, followed by Tukey's post hoc test for multiple comparisons.

The analysis of viewings of spring 2019 archives for P3 courses also showed an increase in the average number of views per lecture for sessions delivered after the suspension of in-person classes between November 2019 and September 2020 (Figures 6A-6D). The highest change in viewing numbers was observed for PHAR406 (36-46 views per lecture) (Figure 6A), PHAR425 (8-14 views per lecture) (Figure 6B), and IC4 (17-41 views per lecture) (Figure 6D).

**Figure 7:**
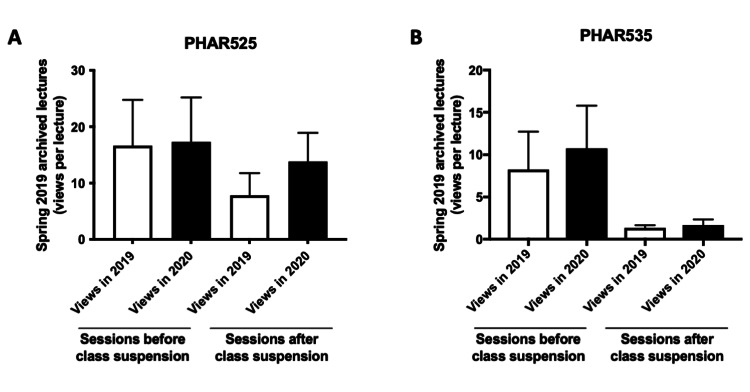
Comparison of the average views per lecture (P4 courses) for spring 2019 archived recordings before and after the suspension of in-person classes. The average number of views per lecture for spring 2019 archived recordings, both before and after the suspension of in-person classes (lectures delivered before and after March 9, 2020, or week 11), was compared at two-time points - November 2019 (2019 views) and September 2020 (2020 views). (A, B) Views per lecture for spring 2019 lecture archives for P4 courses (PHAR525, PHAR535). The bar charts display the average number of views per lecture, presented as mean±SEM. Group comparisons were performed using one-way ANOVA, followed by Tukey's post hoc test for multiple comparisons.

As shown in Figures 7A, 7B, a similar pattern was also noted with P4 courses, where an increase in the average number of views per lecture for spring 2019 archived sessions delivered in the period after the suspension of in-person classes was observed between November 2019 and September 2020. Of note, viewing numbers increased for PHAR525 from 8 to 14 views per lecture (Figure 7A), while no major change was observed for PHAR535 (Figure 7B).

**Figure 8:**
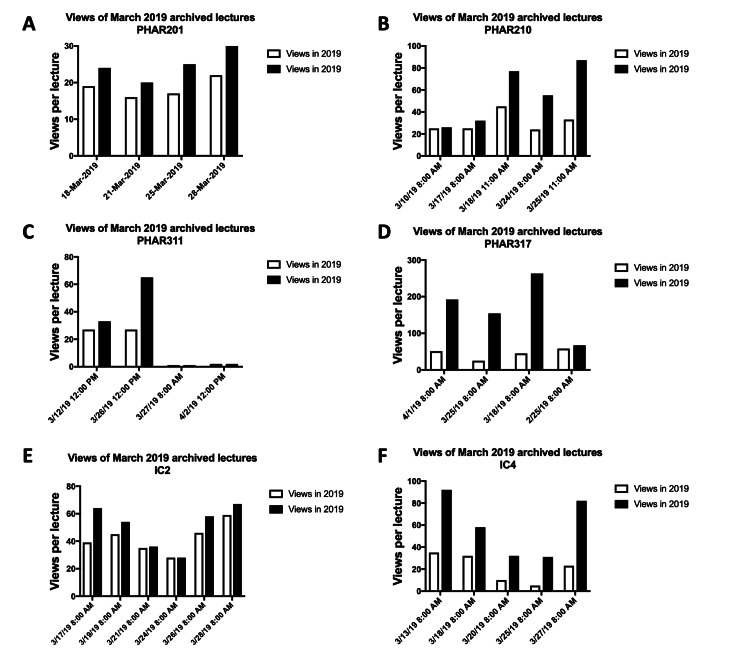
Average views per lecture for spring 2019 archives during the early weeks of the suspension of in-person classes (March 2020). The average number of views per lecture for spring 2019 archived sessions delivered in March 2020, during the early weeks of in-person class suspension, across selected undergraduate pharmacy courses from various levels. Courses include PHAR201 (A) and PHAR210 (B) (P1 courses); PHAR311 (C), PHAR317 (D), and IC2 (E) (P2 courses); and IC4 (F) (P3 course).

The most significant increases in the number of views for spring 2019 archives between November 2019 and September 2020 were observed for lectures delivered in the first three weeks following the suspension of in-person classes, indicating a notable shift in viewing behavior during the transition to remote learning. For instance, the archives of the first three lectures of PHAR317 (P2 course) delivered after the suspension of on-campus classes exhibited a strong increase in the number of views in September 2020 compared to November 2019, rising from 45 to 263 (session of March 18, 2019), from 25 to 154 (session of March 18, 2019), and from 51 to 192 views (session of 1/4/2019), respectively. For IC4 (P4 course), the archive of sessions delivered on March 13, 2019, soared from 35 to 92 views. A similar pattern of increase in the number of archive views of lectures delivered in the first three weeks following the start of distance-based learning was also observed for other professional pharmacy courses across all levels between November 2019 and September 2020 (Figures 8A-8F).

Impact of suspension of on-campus classes on the pattern of VLC viewings by students during the time of the day

To obtain a deeper understanding of the usage patterns of lecture archives by undergraduate pharmacy students, we correlated the frequency of archive viewings with the time of day and conducted a comparative analysis between spring 2019 and spring 2020, as well as examining variations between the periods before and after the suspension of on-campus classes.

**Figure 9:**
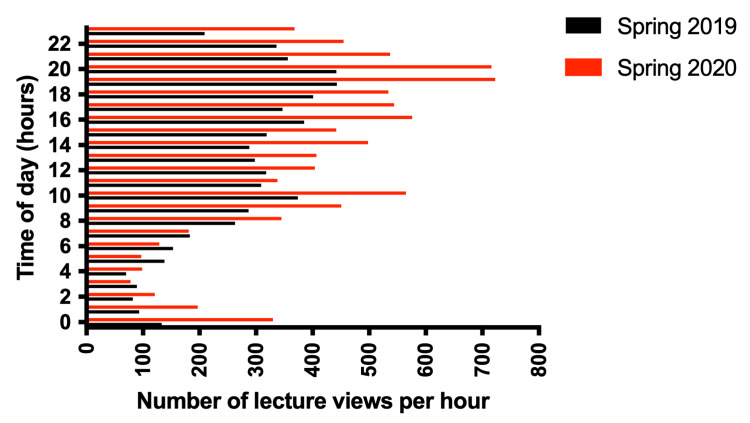
Hourly comparison of lecture archive viewings between spring 2019 and spring 2020. Comparison of the number of lecture archive views per hour of the day between spring 2019 (black bars) and spring 2020 (red bars).

As shown in Figure 9, the analysis of Echo360 lecture archive viewings throughout the whole spring 2019 semester (January 1, 2019, to May 31, 2019) showed that the highest hourly views occurred between 4 PM and 11 PM (2,710 views out of a total of 6,316 views in the semester or nearly 31% of all views in the semester). Throughout the whole spring 2020 semester (January 1, 2020, to May 31, 2020), the highest hourly number of views occurred between 4 PM and 11 PM (4,085 views out of a total of 9,135 views in the semester or nearly 45% of all views in the semester). The peak of views was observed between 6 PM and 9 PM in both spring 2019 (1,286 views out of a total of 6,316 views in the semester or 19.6%) and spring 2020 (1,973 views out of a total of 9,135 views in the semester or 21.6%).

**Figure 10:**
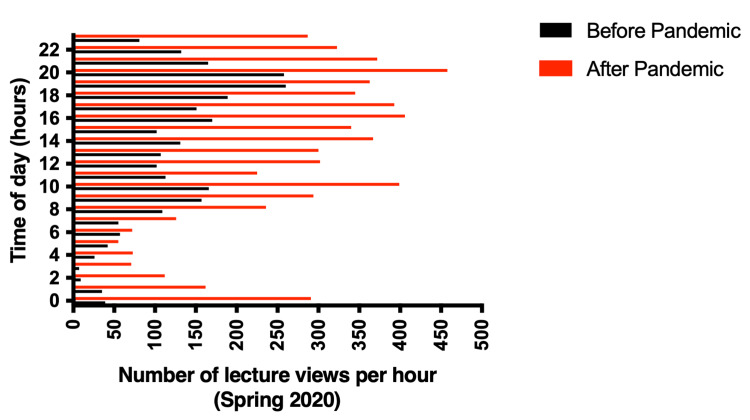
Hourly comparison of lecture archive viewings before and after the suspension of in-person classes in spring 2020. Comparison of lecture archive views per hour of the day in spring 2020, before (black bars) and after (red bars) the suspension of in-person classes (pandemic).

We focused then on the impact of the on-campus suspension of classes on how the students access the Echo360 archives during the time of the day. We analyzed the hourly total number of views before the suspension of face-to-face classes (January 1, 2020, to February 29, 2020) and after that (March 1, 2020, to May 31, 2020). As shown in Figure 10, before the suspension of face-to-face classes, students viewed lecture archives mostly in the afternoon through the evening (4 PM onwards) with a peak between 6 PM (260 views) and 9 PM (258 views). A similar pattern was observed in the period after the suspension of in-person classes where students also viewed the lecture archives more in the afternoon through the evening (after 4 PM) with a peak of views between 6 PM (463 views) and 9 PM (458 views); however, the number of hourly views was much higher in the period post-suspension of classes compared to before, across all hours of the day, with an increase of 1.7-fold during peak time (6-9 PM) (Figure 10).

Impact of assessments on the number of viewings of lecture archives

Table 2 summarizes the number of assignments and assessments and the number of lecture archive views for each week of spring 2020. Prior to the suspension of in-person classes, the number of lecture archive views increased steadily throughout the semester between weeks one and 10 (mid-semester break), with numbers sharply increasing in the weeks when the students were sitting multiple midterms and handing over assignments in the same week (weeks six to nine). Although week 10 was a mid-year break, it showed the highest number of views in the period prior to the suspension of on-campus classes. This was likely because students were expected to take several major midterms immediately after the break in week 11.

After the suspension of on-campus classes (week 11), the number of lecture archive views soared to 879 views per week and continued at this high level for three weeks in a row (weeks 11-13). In the following weeks (14-16), the number of views decreased by around 40% (an average of 526 views per week); however, it stayed at higher levels compared to the period prior to face-to-face class suspension. Of note, week 17 showed another major spike in the number of views (794 views). Week 17 had the highest number of assessments (18 assessments) and also preceded the start of the final exams period from week 18 onwards.

## Discussion

This study aimed to investigate how the COVID-19 pandemic impacted the utilization of Echo360 lecture archives by undergraduate pharmacy students, comparing data from spring 2019 (pre-pandemic) with spring 2020 (during the pandemic). This analysis underscores the pivotal role of lecture archives as a vital support system during the rapid shift to remote learning due to the COVID-19 pandemic. Our methodology of analyzing system-generated viewing statistics facilitated an objective comparison between the pre-pandemic and pandemic periods, mitigating any biases associated with self-reported data.

Our analysis indicates a 45% rise in lecture archive views in spring 2020 relative to spring 2019, with a significant surge in views subsequent to the cessation of in-person classes. The average weekly views increased markedly, highlighting the critical role of archived lectures in delivering important academic support when in-person instruction was unfeasible. During this transitional phase, access to archived lectures emerged as a crucial resource for students, especially in the weeks immediately following class suspensions, when live lectures had not yet completely resumed online. The availability of archived lectures facilitated continuity, enabling students to sustain a learning rhythm and engage in self-study during a time of uncertainty.

After the emergency switch to distance learning in the spring of 2020, all lectures stopped being recorded via the Echo360 system as the process requires delivery of the lecture inside the classroom to use the recording hardware. Therefore, we postulated that the increase in the number of views in spring 2020 compared to spring 2019 and the augmented number of views in the period post-suspension of on-campus classes in spring 2020 could have been driven by an increase in the number of views of spring 2019 archived lecture recordings. To verify this, the number of views per lecture was analyzed for spring 2019 archives at two-time points (November 2019 and September 2020). Data for November 2019 were already collected for a previously published study [13]. The analysis showed that for all courses studied and for all professional pharmacy undergraduate levels (P1-P4), the average number of views per lecture was higher in September 2020 compared to September 2019, indicating an increase in views of spring 2019 archives by students during spring 2020. This contrasts with observations from our previously published study, where lecture capture viewings were followed across undergraduate pharmacy courses for three consecutive academic years, showing that the system was used more by junior students (P1 and P2) compared to students in advanced years (P3-P4) [13]. Previous studies also highlighted that students tend to view more lecture capture recordings at the beginning of the academic year (fall semester) and level off thereafter; however, in the current study students viewed recordings more in spring 2020 further harnessing the impact of the pandemic on the increase in use [10,13].

From the analysis of Echo360 archive views per week in spring 2020, it is shown that the number of views per week increased steadily throughout the semester; however, a very sharp increase was observed during the first three weeks following the suspension of in-person classes (weeks 11-13). This was further illustrated by looking at the number of views for previous year archives of sessions taking place in the first three weeks of the suspension of face-to-face classes (weeks 11-13). We observed that courses with calculations and integrated course series witnessed the highest jumps in the number of views per lecture for the sessions delivered in the first three weeks following the suspension of in-person classes. For example, PHAR317 (pharmacokinetics; P2 course) exhibited a strong increase in the number of views in September 2020 compared to November 2019 for archives of the first three sessions in weeks 11-13 (45-263 views for the session of March 18, 2019; 25-154 views for the session of March 18, 2019; and 51-192 views for the session of April 1, 2019). This pattern was also observed for many other spring courses, including the integrated course series, IC2, and IC4. Altogether, this highlights the importance of having archived lectures from the previous cycle (Spring 2019) available to students. These recordings supported their learning during the emergency transition to distance-based learning, particularly in the first three weeks of class suspension, while instructors were shifting to live teaching using video-conference platforms such as WebEx, Blackboard Collaborate Ultra, Microsoft Teams, or Zoom [18].

We mapped the number of Echo360 lecture capture views per week in spring 2020 to the number of assignments and assessments delivered. The pattern of lecture recording viewings prior to the suspension of on-campus classes followed a more or less predictable mode where students steadily viewed the archives as the semester weeks progressed and then peaked in weeks where multiple assessments were scheduled (weeks six to nine). The number of views peaked further in week 10, although this was the mid-semester break because students may have been preparing for midterms scheduled after in week 11. As mentioned above, the number of weekly views dramatically soared in the first three weeks (11-13) after the suspension of classes, despite the fact the College postponed all midterms and assignments scheduled during this period to allow time for online live teaching to be organized and assessment strategy for all courses to be revised and adapted to online-based learning [13]. This underscores the fact that the increase in these three weeks was not primarily motivated by assessments but rather by a need for students to support their learning while online-based learning is organized. In subsequent weeks (14-16), the number of weekly views of lecture archives declined by nearly 40% compared to the first weeks of the pandemic; however, it stayed at higher levels compared to the period prior to face-to-face class suspension caused by the COVID-19 pandemic. It is anticipated that students during this period also started viewing new lecture recordings delivered using video-conference platforms posted on the VLE. This steady high viewing number of archives coincided with the resumption of assignments and assessments from week 15 onwards including the objective structured clinical exam (OSCE), a major exam sat by final year students (P4). Another spike in the weekly views was observed in week 17 (794 views). This week had 18 assessments across all four professional years (the highest number in the semester) and also preceded the final exams period (week 18 onwards). Similarly, the sharpest increase in weekly views that was observed in the previous academic year (spring 2019) was in week 19, immediately before the start of the final exams period. This finding is consistent with that of other studies which found that students viewed lecture capture recordings the most in the weeks before assessments [19-22]. In a study by Copley et al., students found lecture capture recordings most useful when revising for exams [23]. This further strengthens the importance of lecture archive viewings to support students’ preparation for assessments.

Another important factor that has been evaluated in the current study was the impact of the suspension of in-class lectures on the way students accessed the lecture archives during the day. The analysis of the hourly views between spring 2019 and spring 2020 revealed that students continued to access the Echo360 lecture archives in a similar pattern during the day with most views occurring after 3 PM and peaking between 6 PM and 9 PM despite the number of views being strongly higher in spring 2020 compared to spring 2019 at all hours of the day. When the impact of COVID-19-related in-person class suspension was considered for spring 2020, it was found that the pattern of accessing the system by the students was not affected by the switch to distance-based learning, where the students viewed the archived lectures after hours with a peak of views between 6 PM and 9 PM, although the number of hourly views soared at all times of the day after the suspension of on-campus classes compared to the period before. Although previously published studies have looked into the frequency of accessing recordings [21,24], Akiyama et al. reported that students preferred to access recordings between 6 PM and midnight, coinciding with the findings from the present study [25]. However, there are currently no studies that have reported such detailed usage patterns within health education, and we believe our finding adds great value to the current literature.

The COVID-19 pandemic has highlighted the essential requirement for preparedness in higher education to manage future disruptions, including emerging risks such as monkeypox or new strains of COVID-19. This necessity is corroborated by numerous research in the literature. Crawford built a preliminary higher education pandemic response model by examining reactions to COVID-19 and prior pandemics. They suggested four phases of pandemic response as follows: fast adaptation, enhancement, consolidation, and restoration. This concept underscores the significance of readiness and flexibility in higher education institutions [26]. Moon et al. demonstrated the necessity for enhanced crisis management in higher education, informed by the experiences of Australian academics. The University of Sydney's reaction to the pandemic highlighted good communication tactics and collaboration [27]. Ismail and Dawood contended that the insights acquired from the COVID-19 pandemic had enhanced educational institutions' preparedness for future outbreaks such as monkeypox [28]. Moreover, Hirani et al. emphasized the necessity of sustained alertness and readiness in educational environments, using insights gained from COVID-19 [7]. These studies together indicate that the higher education sector acknowledges the necessity of formulating flexible response methods, resilient communication networks, and adaptable learning models to proficiently address future disturbances. Higher education institutions must proactively include TEL and lecture recording infrastructure in their programs. This readiness can guarantee minimum interruption to educational delivery if conventional in-person instruction is hindered. The archived lecture materials from spring 2019 efficiently addressed content delivery deficiencies in spring 2020, demonstrating the significant use of such solutions in crises. Students observed that access to these archives greatly facilitated their learning during the shift to distance learning [18].

Integrating TEL and systematic lecture recording into the academic environment offers advantages that surpass mere disaster readiness. Access to recorded lectures improves the student learning experience by enabling review of intricate material at their own pace, which is especially advantageous for courses necessitating detailed calculations or substantial foundational knowledge (e.g., medicinal chemistry, pharmaceutics, and pharmacokinetics). It also benefits students with special needs, thus ensuring a more inclusive learning environment. Reliable access to recorded sessions offers students enhanced flexibility, allowing them to review difficult ideas while preparing for evaluations, a primary application of lecture video recordings demonstrated in prior research [19-23]. During crises like the one experienced during the COVID-19 pandemic, increased demand for viewing lecture video recordings might result in technical difficulties, mostly owing to system capacity constraints. Our experience indicates that the heightened utilization of the Echo360 video recording technology presented some technological challenges. Despite Echo360 employing external servers for video storage, alleviating capacity concerns linked to our VLE (i.e., Blackboard 9), students accessed the recordings through the VLE. The dependence on Blackboard resulted in system overload, especially during peak usage periods when students utilized the VLE for additional activities, including accessing lecture notes, submitting assignments, and conducting assessments. Elevated system usage occasionally surpassed capacity thresholds, resulting in connectivity problems and sporadic access disruptions; certain students encountered connection losses or login failures attributed to these demands, including periodic internet or Wi-Fi instability. These difficulties need technological modifications and supplementary assistance to ensure consistent system performance [18].

The incorporation of TEL and video lecture recording systems provides faculty with a robust mechanism for self-evaluation and enhancement of their teaching strategies. Instructors can improve their delivery, modify teaching approaches, and pinpoint opportunities to boost student engagement by analyzing their recorded lectures. These archives enable peer observation of pedagogical methods, an essential method for quality enhancement and faculty development. New faculty members might gain insights by examining the teaching methodologies of seasoned colleagues, promoting a culture of collaborative learning and pedagogical advancement. Furthermore, the existence of a systematic, accessible archive of recorded lectures offers essential resources for continuous professional development initiatives and orientation programs for new instructors [29,30].

The availability, at our college, of a cohesive virtual learning environment (VLE) equipped with an effective VLC system many years before the pandemic mitigated the effects of the swift shift to remote learning. The familiarity of both students and staff with the Echo360 archive system, which they had regularly utilized across the curriculum for several years, facilitated a smoother and more effective transition to online instruction than would have otherwise been possible [13]. This previous experience provided students with a self-directed learning resource during the early transition phase, allowing instructors to concentrate on modifying live content delivery via video conferencing technologies like WebEx, Blackboard Collaborate Ultra, Microsoft Teams, and Zoom [18].

An examination of viewing patterns during spring 2020 indicates students' versatility in utilizing archived resources, with maximum access occurring post 3 PM, consistent with previous research highlighting students' inclination towards nighttime study [21,25]. This viewing pattern, along with prior usage trends, indicates that the transition to distance learning did not markedly change students' choices for after-hours study, underscoring the necessity of accessible, flexible learning resources.

We recommend that higher education institutions establish a systematic methodology for video lecture capture (VLC), recording practices, and archiving protocols to prepare for potential future educational interruptions. The results indicate that archived lectures were essential for students during the pandemic and also bolstered a strong learning infrastructure in conventional conditions. By maintaining a repository of lecture archives separate from the VLE storage limitations, institutions may ensure the longevity of these resources without burdening other digital assets, such as lecture notes and assignments. Echo360 and comparable platforms guarantee the prolonged accessibility of preserved resources, providing continuity and stability in academic assistance. However, it is important to highlight that the implementation of video lecture recording in conventional educational settings prompts valid fears regarding student attendance. As discussed in our introduction above, there is apprehension among some faculty that easily accessible recordings may discourage students from attending live classes. However, research studies in this area did not all conclude this. While certain studies indicate a decrease in attendance when VLC is available, others report no significant effect, suggesting that factors beyond availability, such as the level of interactivity, student engagement, and perceived relevance of in-person sessions, play significant roles [15-17]. Effective course design and engaging delivery methods can help mitigate the risk of over-reliance on recorded material, maintaining appropriate student attendance and active participation. Moreover, our university implements a stringent attendance policy, requiring students to attend a minimum of 75% of lectures to prevent course failure. This policy diminishes the probability of students depending solely on recordings rather than attending in person.

Lecture recordings and archives may also raise legitimate concerns about privacy and copyright. At our institution, the video lecture recording system exclusively records in-person, face-to-face lectures, with instructors retaining full discretion over the decision to record. This in-class recording configuration mitigates the possibility of unauthorized recordings by students, a concern often present in online teaching platforms (e.g., WebEx, Blackboard Collaborate Ultra, Zoom). The device solely records the instructor, and the educational content projected on-screen, omitting any visuals or identifiers of students, so safeguarding attendee privacy. Instructors possess complete authority to halt or terminate the recording at their discretion, with Echo360’s technology connected to classroom hardware that can be deactivated if desired, offering flexibility for instructors who choose not to record. Moreover, recorded lectures are securely housed on Echo360’s external servers and can only be accessed via the university’s secure virtual learning environment (VLE/Blackboard), with access limited to students enrolled in the relevant course. This regulated access prevents unauthorized dissemination beyond the university, safeguarding both the confidentiality of classroom discussions and the copyright of educational resources.

Some limitations should be considered in this study, particularly regarding the technical constraints associated with the Echo360 tracking system for views. While it is expected that the majority of views are from students, the system is unable to track the role of users (e.g., students or faculty), and anyone with access to courses on Blackboard 9.1 VLE is, therefore, able to view the archives. In addition, it is not possible to identify whether the views emanated from different individual users or if they were rather multiple views from a single user. The views tracking system does not provide information on how long a user has viewed the lecture recording. Furthermore, our research focused on a single institution (Qatar University) and specifically analyzed the utilization of lecture recordings by undergraduate pharmacy students. This concentrated approach provides significant insights into pharmacy education, while it may restrict the applicability of our findings to other medical and health specialties within our university.

## Conclusions

This study emphasizes the critical importance of TEL and VLC systems in strengthening the resilience of higher education institutions. The methodical deployment of the Echo360 video lecture recording technology at our university, especially in the College of Pharmacy, has shown its significance in enabling a smooth shift to remote learning. The decade-long integration of this technology at our college equipped students and instructors with the requisite experience and resources to transition rapidly from on-campus to remote education, illustrating the system's efficacy in preserving educational continuity without disruption. However, further research is important to comprehensively understand the students' needs and motivations for utilizing Echo360 during the COVID-19 pandemic and beyond during conventional conditions. Future research may encompass quantitative surveys and qualitative interviews to investigate, in detail, the precise aspects influencing students' dependence on recorded lectures, including accessibility, content review preferences, and flexibility in learning styles. Furthermore, investigating other innovative TEL methods, including interactive video quizzes, adaptive video learning pathways, or the incorporation of augmented reality components, may significantly improve the educational experience. Longitudinal research examining the effect of video recordings on learning outcomes across various student demographics (e.g., academic level, major, learning preferences) would yield significant insights into the impact of lecture recording archives on engagement and performance over time. Enhanced comprehension of the factors influencing TEL utilization can guide future enhancements, guaranteeing that stored lectures remain essential for student learning, assessment preparation, and instructional quality in both conventional and disrupted educational environments.

As higher education institutions confront possible challenges from global future health crises, the adoption of a comprehensive TEL strategy is essential. This encompasses the long-term storage of lecture archives and the utilization of web-based solutions for content recording and dissemination. These solutions not only augment the student learning experience and facilitate innovative pedagogical methods but also bolster institutional preparedness, guaranteeing that education remains accessible and of high quality amid any future disruptions.
